# Women With Obesity Are Not as Curvy as They Think: Consequences on Their Everyday Life Behavior

**DOI:** 10.3389/fpsyg.2019.01854

**Published:** 2019-08-16

**Authors:** Isabel Urdapilleta, Saadi Lahlou, Samuel Demarchi, Jean-Marc Catheline

**Affiliations:** ^1^Department of Psychology, Paris 8 University, Saint-Denis, France; ^2^Department of Psychological and Behavioural Science, London School of Economics and Political Science, London, United Kingdom; ^3^Paris Institute for Advanced Study, Paris, France; ^4^Digestive Surgery Department, Delafontaine Hospital, Saint-Denis, France

**Keywords:** women, obesity, perceived body, represented body, everyday behavior

## Abstract

Two studies explore the impact of body size on daily life activities of women with obesity. In the first study, ethnographic techniques (first-person perspective video recordings) and subsequent interviews based on the video recordings were used. Results showed atypical behavior of women with obesity and ex-obese women related to memories of embarrassing experiences regarding personal body size (sitting, passing doors sideways, over-careful navigation in public space, and choosing clothes sizes too large.) Women with obesity seem to behave as if they thought they had a larger body than it actually was. These atypical behaviors are related to memories of embarrassing experiences regarding personal body size and stigma. Overweight women exhibit the same behavior but to a lesser and less systematic degree. In the second study, the represented (imagined) body size was compared to the perceived (in a mirror) body size with digital morphing techniques. In the mirror condition, the perceived image is accurate, while in the absence of a mirror women with obesity overestimate their body size by about 30%. Moreover, overestimation of imagined body size increased according to the weight status. Finally, women who had bariatric surgery had poorer estimates than women who had not. This would result of being continuously reminded of obesity and its stigma by daily embarrassing experiences, by being confronted with an environment designed for normal weight (e.g., narrow seats, turnstiles etc.) that makes obesity salient. We suggest that body size overestimation is a case of accentuation where things that matter are perceived bigger. These results could also been explained by the allocentric lock theory.

## 1. Introduction

Since the 1980s, there has been a dramatic increase worldwide in obesity rates (Finucane et al., 2011; Flegal et al., 2012). Based on the latest estimates in European Union countries, 30–70% of adults are overweight and 10–30% of adults are obese (World Health Organization, 2017). Obesity is one of the greatest public health challenges of the twenty-first century since high body mass has been shown to be associated with multiple domains of poorer health and related quality of life (Doll et al., 2000; Taylor et al., 2013; Latner et al., 2014).

A systematic review (Kroes et al., 2016) of US literature demonstrated that obesity but also overweight status are associated with poorer health related quality of life than normal weight. In Europe (England), Søltoft et al. (2009) investigated the relationship between body mass index (hereafter BMI) and health-related quality of life, and potential differences between men and women. Results show that BMI is negatively associated with health-related quality of life for both underweight and obese individuals. But at higher BMI values, women reported less health related quality of life than men.

Health-related quality of life can be classified into physical and mental or psychological components. Regarding physical components, increased weight is associated with significant health impairment and medical comorbidities (Bray, 2004; Lawrence and Kopelman, 2004; Mitchell et al., 2015). For example, the link between obesity or overweight with an increased risk of cardiovascular disease is well-established (Poirier and Eckel, 2002; Poirier et al., 2006; Caleyachetty et al., 2017). Many other conditions such as type 2 diabetes are more prevalent in overweight and obese individuals (Slagter et al., 2015; Boles et al., 2017). Several cancers are also more prevalent in high body mass individuals (Calle and Kaaks, 2004; Calle and Thun, 2004; Abdulhussein and Amin, 2018). Regarding mental or psychological components, obesity is sometimes associated with depression (Onyike et al., 2003; Faulconbridge et al., 2018) and social discrimination (Puhl and Brownell, 2001) because of the shame and stigma attached to obesity (Puhl and Heuer, 2009; Brewis, 2014; Westermann et al., 2015; Spahlholz et al., 2016; Vartanian et al., 2016). Myers and Rosen (1999) asked obese people to list stigmatizing situations they had encountered then noted the frequency with which they encountered each form of stigmatization. Respondents reported their own experiences with stigmatization in an open ended format. Participants included clinical (consecutive severely obese patients in a gastric bypass surgery program), and non-clinical samples of obese persons (members of an electronic mail list service). These two samples of obese persons were asked to complete an open-ended questionnaire on stigmatizing situations. A total of 50 stigmatization experiences were identified. Authors concluded that “being overweight can cause problems for people, not only medically, but in social situations as well” (p. 223). The three most frequent stigmatizing situations faced were comments from children (“A child coming up to you and saying something like, ‘You're fat”'), other people making negative assumptions about the obese person (“having low expectations of you because of your weight.”), and encountering physical barriers (such “not being able to sit into seats at restaurants, theaters, and other public places” or “not being able to find clothes that fit.”).

Concerning weight status, a review by Puhl and Brownell (2001) investigated years of research examining bias toward overweight and obese individuals. They found that weight discrimination and negative attitudes toward overweight and fat individuals are apparent across various environments (see also Puhl and Heuer, 2009, 2010; Flint et al., 2016). Even, studies using daily diary assessments report much higher rates of stigma experiences in obese than overweight individuals. In fact, as weight increases, weight-related stigma experiences increases (Vartanian and Novak, 2011; Spahlholz et al., 2016). For example, Myers and Rosen (1999) show that individuals within the severely obese range of body mass index (BMI) of 40 kg/m^2^ or greater, reported more stigmatizing situations than those with a BMI <40 kg/m^2^. Concerning gender status, there are mixed findings in the literature on whether men and women experience weight based stigma. Some studies find no difference in reported rates (Puhl and Brownell, 2006; Vartanian and Novak, 2011; Jackson et al., 2014; Vartanian et al., 2014; Vartanian, 2015), while others have found women experience higher rates of weight stigma than men (Andreyeva et al., 2008; Puhl et al., 2008; Fikkan and Rothblum, 2012; Spahlholz et al., 2016). In fact, men and women experience weight stigma at different levels of body weight (Himmelstein et al., 2018). Women report weight discrimination at lower levels of excess weight than men. For example, men tend to report considerable stigmatization at a BMI of 35 or higher, whereas women report experiencing notable increases in weight discrimination at a lower BMI of only 27 (Spahlholz et al., 2016). Among women, reports of weight stigma tend to follow a linear pattern, with women experiencing more weight stigma as they move into higher BMI categories (Hansson et al., 2010; Judge and Cable, 2011).

People who are exposed to discrimination in their environment may be at risk for body image problems (Myers and Rosen, 1999; Cash, 2004). Indeed these negative weight stigmatization messages can become internalized, reflecting weight self-stigma (Durso and Latner, 2008; Lillis et al., 2010). Weight self-stigma is a construct involving negative emotions and beliefs about being overweight or obese and fear of enacted stigma (i.e., perception of being discriminated against and of belonging to a stigmatized group; Link and Phelan, 2001; Bos et al., 2013). Note that these findings seem to be particularly relevant as weight loss may not necessarily diminish weight-related stigma (Milkewicz et al., 2004; Fardouly and Vartanian, 2012; Latner et al., 2012). Authors have studied how currently overweight, formerly overweight, and never overweight individuals differ in a range of eating and body image measures and report residual body image problems following weight loss. People who have been overweight in the past do not ultimately obtain the same positive body image when they lose weight as someone who has never been overweight (Cash et al., 1990). These observations may be interpreted as being the result of memories of shame and discrimination, but also as *phantom fat* phenomenon (Cash et al., 1990). The phantom fat is a phenomenon where people lose weight and yet still represent themselves as with overweight/obesity; the body has shrunk but the representation has remained the same.

Several constructs have been proposed and studied in the literature. One can distinguish between body image and body schema, i.e., the body as an intentional object of consciousness vs. a non-conscious performance of the body (Gallagher, 1986). Riva (2018) proposed to consider the integration of six different aspects of embodied experience into a single matrix of body representation. Especially interesting for our purpose are the egocentric and allocentric aspects (embodied perspective in the subject as a reference of experience vs. originating in the environment including social). These two aspects resonate with the Meadean notions of the “I” (experienced by the acting subject) vs. the “Me” (socially constructed) (Mead, 1934). Impairment in integration of the various dimensions of the body could be reflected in many symptoms of eating disorders (Riva, 2014, 2018; Serino et al., 2015). The allocentric lock hypothesis (Riva and Gaudio, 2012; Riva, 2018) suggests that defective ego/allocentric integration locks subjects in an external, enduring, body image.

The focus of this article is to explore the impact of weight/size on daily life. How do women with obesity move and live with their bodies in social space? Are there differences between normal weight, overweight, and obese? What happens after bariatric surgery? The paper presents two studies, an ethnographic one and an experimental one, both using cutting edge techniques. In study 1, a sample of Parisian women record their own mundane activity from the first-person perspective with a miniature wearable camera. The tapes are coded to compare the behavior of the participants to standard cultural behavior in situations known to be challenging for people with obesity: choosing seats in public transport, passing doors, navigating public space, choosing a garment in a shop. We used ethnographic methods to observe and discuss how women (normal weight to obese persons) behave when these occasions occur. These situations refer to physical barriers, the most frequent stigmatization situation identified by Myers and Rosen (1999) in their inventory. These physical barriers seem to be encountered about 18 times per day by individuals with overweight status and obesity (Vartanian et al., 2014). What is their impact on behavior and representations? How does this vary with BMI? To investigate the possible causes of the atypical behavior documented in study 1, we then test in study 2, (a) whether women of larger size have an overestimated representation of their body size, and to what degree and (b) whether this overestimation, if any, is an overestimation in the perception of their own body (with their senses, as is the case in phantom limbs) or in the representation of their body (in their imagination).

## 2. Study 1. Observing Behavior in Natural Settings

Pilot ethnographic study (Urdapilleta and Lahlou, 2012; Urdapilleta et al., 2017) showed that persons with a high BMI adopt specific behaviors in situations where body size matters (e.g., when sitting on public transportation). It also suggested that behavior is not simply dependent on actual body size, but also on previous personal history (see also Hamlet et al., 2016). Indeed, participants who had recently undergone drastic body size reduction (through bariatric surgery) seemed to continue to behave as they did when their body was large, as if some larger phantom body (Cash et al., 1990) remained in their mind and habits. The issue therefore appears more complex than one of larger bodies being challenged by the affordances (Gibson et al., 1982; Gibson, 2014) of a built environment designed for individuals with normal weight, such as narrow seats. These little details of everyday life contribute to persons with a high BMI being repeatedly identified and stigmatized as obese, with all the detrimental consequences associated with stigma (Hinman et al., 2015; Pearl and Puhl, 2016).

To address the problem, we explore not only what individuals with obesity actually do, but also how they experience situations. This first study investigated this issue by following the daily activities of women in their mundane life, thus going beyond diaries to observe actual behavior *in situ* and collect detailed data about participants' experiences through self-confrontation with their first-person perspective recordings. We compared the mundane behaviors of women with different body sizes and body size histories in order to better understand what experiences and representations drive their behaviors, especially those considered outside of the norm by the standards of the local culture.

### 2.1. Methods

#### 2.1.1. Participants and Procedure

The study included 14 French women aged 20–48 (*M* = 28.36, *SD* = 5.54). They were divided by physiological characteristics into seven groups of two, according to weight status but also to whether they had bariatric surgery (*n* = 6) or not (*n* = 8). At the time of the study, the non-surgery group (hereafter NS) included two ex-obese women who had class 3 obesity (BMI 40 and above), two women with class 1 obesity (30 > BMI < 34.9), two women with overweight status (25 > BMI < 29.9) and two normal weight women (18.5 < BMI < 24.9). The surgery group (Hereafter S) included two women with obesity class 1 women who had surgery 3 months ago, two women with overweight status who had surgery 8 months ago, and two women with overweight status who had surgery 4 months ago. Table 1 provides the participants' characteristics (all names were changed). This sample was selected to provide enough diversity in body size and body-size history to allow comparing data obtained using detailed qualitative, behavioral, and experimental methods.

**Table 1:** Participants' characteristics.

**Participant**	**Age**	**Current status**	**Current BMI**	**BMI before surgery**	**Weight loss**	**EBMIL**
Laura	33	NS-O3	54.7			
Linda	24	NS-O3	60.3			
Dorothy	26	NS-O1	32.2			
Deborah	25	NS-O1	34.7			
Carol	22	NS-OW	25.1			
Carla	28	NS-OW	28.4			
Mary	27	NS-NW	21.6			
Margaret	36	NS-NW	23.8			
Anita	20	S-O1-3	33.9	39.8	17	39.8
Anna	27	S-O1-3	34.2	42.5	21	44.0
Suzan	38	S-OW-8	29.0	42.8	38	77.4
Sarah	24	S-OW-8	28.4	41.9	39	80.0
Karen	32	S-OW-4	31.1	41.5	30	62.8
Kerry	35	S-OW-4	29.3	38.6	25	67.7

*NW refers to Normal Weight women, OW refers to overweight Women, O1 is women with Obesity class 1, and O3 is women with Obesity class 3. NS prefix refers to women who had no surgery, and S Prefix refers to women who had surgery. EBMIL is the percentage of excess BMI still needed to be lost for the participant to be considered out of the “overweight” classification (Deitel and Greenstein, 2003): 100 - [(follow-up BMI-25/Beginning BMI-25) × 100]*.

Participants' education level ranged from Business and Technology Education Council (BTEC) First Diploma to Masters' degree in all groups. Women were recruited through the hospital where they registered for surgery, through a call for volunteers among the cohort of patients who had already registered for surgery, and through snowball sampling, starting with a convenience subsample of university employees.

The Subjective Evidence-Based Ethnography (SEBE) was used. SEBE is a digital ethnographic technique that comes in three steps: (1) capture of actual activity in natural settings by the participants themselves, with a wearable, unobstrusive (7 g) miniature camera called subcam (Lahlou, 1999). (2) replay interview where participants are confronted with their tapes and comment it to the researcher. At this stage, the researchers can not only listen to the participants interpretation (emic), but also test if the way they translate these interpretations into their own words (etic) are validated by the participant (Kottak, 2005; Xia, 2011). As the first-person perspective recordings re-immerse the participant in her own perception action loop, the participants access episodic memory (Tulving, 2002) and re-enact the situation: remembrance of actions, emotions, and intentions is outstanding. The technique, and especially its stringent ethics guidelines (Lahlou, 2011, 2018; Lahlou et al., 2015).

##### 2.1.1.1. Phase 1. Capture of actual behavior (subcam)

Participants transparently recorded what they did using a wearable, light, and discreet miniature video camera worn on a pair of glasses, called a subcam. Subcams provide first-person perspective recordings of the visual field with wide-angle lens. Subcam recordings radically differ from classic films, even from the cinematographic point-of-view shot, as the camera follows the rapid head movements of the wearer and therefore attentional focus.

Participants were instructed to take public transportation (e.g., metro, train), to shop for clothes, and to try on at least one garment (e.g., coat or jacket). They were alone and free to choose times and places. Nevertheless, a researcher stayed in the vicinity in case the participant needed support and called on her mobile phone (out of sight but close enough). The subcam recordings lasted between 60 and 98 min (*M* = 68.20, *SD* = 14.08).

##### 2.1.1.2. Phase 2. Replay interviews (RIWs)

Participants were interviewed as they replayed their own subcam recordings. Based on the findings of the pilot study, we clipped excerpts when participants chose a seat, went through turnstiles or doors, or tried on a garment. Replay interviews took place with two psychologists and lasted between 74 and 110 min (*M* = 83.20, *SD* = 15.50). RIWs were video-recorded and fully transcribed. Because the films contain rich situated visual, auditory, and kinetic cues, participants recalled their mental states (goals, interpretations, and even feelings) at the time they acted with pristine accuracy and could verbalize them. Participants apparently re-enacted the situation as they watched their own first-person perspective recordings. Similar effects of situated interviewing on recall have been described in embodied cognition literature (Dijkstra et al., 2007; Barsalou, 2009), especially regarding the positive influence of kinetic cues. The clips were explored with participants during the RIWs, with a focus on the reasons for action and the feelings experienced by participants.

#### 2.1.2. Ethics

The research followed all applicable institutional and governmental regulations concerning the ethical use of human volunteers. The protocol followed the guidelines of the British Psychological Society and the SEBE guidelines, which add specific safeguards against possible issues of video material. This includes a moratorium period, during which the participants keep their film before the researchers see it and consider whether they want part or all of the footage erased. The protocol was validated by the Social Psychology Ethics Committee at the London School of Economics and Political Science (Houghton St, WC2A 2AE, London, UK). All participants were volunteers and were free to withdraw at any stage of the study. No remuneration was given. Finally, all participants were given a special telephone hotline in case they had second thoughts or questions. Written informed consent was signed by the participants.

### 2.2. Data Analysis

Extracts (clips) were initially selected for analysis if they contained occasions that participants in the pilot study identified as problematic for women with obesity (i.e., choosing a seat, going through a door or turnstile, trying on a garment). Then all data were coded by 10 women aged 22–45 (*M* = 29.36, *SD* = 7.54) who had not participated in any of the two studies (hereafter raters). These are normal-weight women living in Paris area. They were attending adult education in psychology and the coding work was done as part of their training.

**Figure 1:**
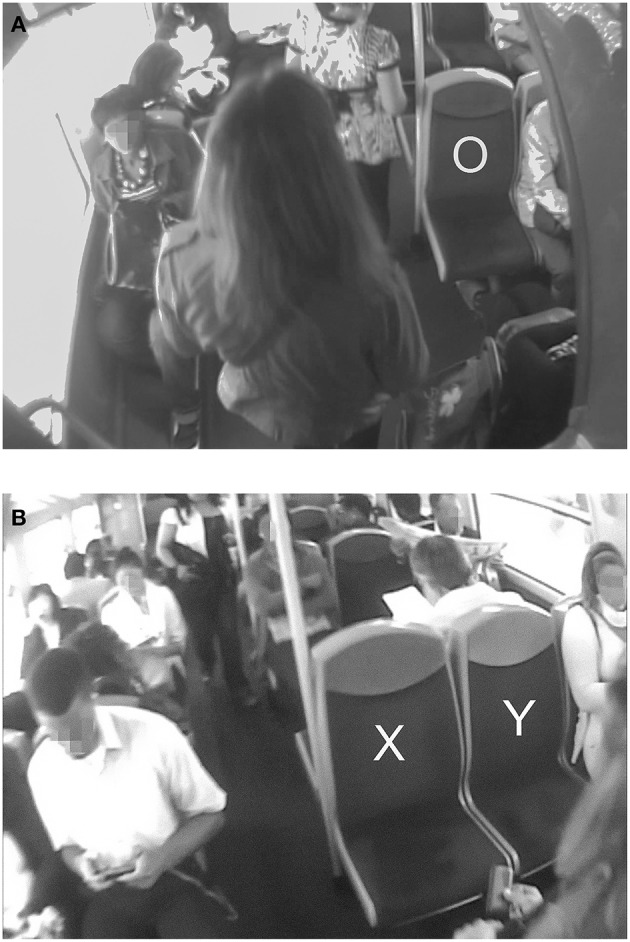
Examples of choosing a seat. Mary does not take the seats marked O **(A)** or Y **(B)**, and walks to the next row to take a seat marked X **(B)**. The participants to the study have given their agreement in writing for the publication of images from their subcam recordings, such as the above, provided they are not individually identifiable on the picture. Images have been blurred to ensure anonymity.

We relied on the analysts' native knowledge of local culture (i.e., contemporary France, the Paris area, middle-class adults) to code the behavior based on standard cultural expectations. For example, social conventions assume that doors and turnstiles are passed through frontways, and that people know their clothing size within an accuracy of plus or minus one size. Another example is that, in a metro car in Paris, local social conventions assume that one should not sit close to another person if there is a free seat available nearby that leaves more interpersonal space. As an illustration, choosing the seat marked with an X, rather than the one marked with an O, as Mary does in Figure 1A, appears perfectly normal (something Parisians would be expected to do), while choosing the seat next to it, marked Y (Figure 1B), would appear unusual according to the local conventions of proxemics, to maintain as much personal distance as possible (1.5–4 feet, according to Hall et al., 1968). For this reason, this behavior (sitting on seat X) can be considered typical in this regard, as containing nothing remarkable, and was coded T (typical). However, it is not necessary to sit further away than the requirements of personal distance dictate, so the closest seat meeting this distance-related requirement will usually be taken.

On the other hand, crossing the entire car to obtain a seat with no neighbors while there are many other closer satisfactory typical choices of empty seats with no neighbor is unusual behavior compared to the cultural norm, and therefore coded atypical. This is what Dorothy did: entering a metro car while there were still many free seats, Dorothy chose to go right to the end of the car to sit on a seat that was on its own (a row of one), with no neighbor, a seat designed for passengers with large items of luggage or baby buggies. There were four free seats (two rows of two) that were closer, and a natural choice, but Dorothy did not sit there. On her way, Dorothy passed several typically acceptable “free seats,” and also persons already sitting on such typical seats, her behavior demonstrating she was pickier than the other passengers. Therefore, Dorothy's behavior here can be considered atypical and was coded so (“AT”). Sometimes behaviors seemed ambiguous and were coded with a question mark (?). Raters coded independently the data, and defined themselves, individually, what they considered typical or atypical behavior. We did not give them a specific coding guide.

### 2.3. Results

Table 2 summarizes the analysis of the subcam recordings and RIWs with the participants.

**Table 2:** Number of occurrences attributed for the 10 ratings for the subcam recordings and replay interviews with participants.

**Participants' name and status**	**Situation**											
	**Sitting on public transportation**			**Going through turnstiles and doors**			**Navigating public spaces**			**Choosing clothing size**		
	**T**	**AT**	**?**	**T**	**AT**	**?**	**T**	**AT**	**?**	**T**	**AT**	**?**
Laura (NS-O3)		8	2		9	1		8	2		10	
Linda (NS-O3)		10			10			9	1		10	
Dorothy (NS-O1)	1	9			10			10			10	
Deborah (NS-O1)		10			10			10			10	
Carol (NS-OW)	9		1	8			8		2		8	2
Carla (NS-OW)	9		1	10			10				8	2
Mary (NS-NW)	9		1	9		1	10			9		1
Margaret (NS-NW)	9		1	10			10			10		
Anita (S-O1-3)		10			10			10			10	
Ana (S-O1-3)		8	2		10		2	8			10	
Suzan (S-OW-8)	9		1	10			9		1		10	
Sara (S-OW-8)	10			10			1	7	2		9	1
Karen (S-OW-4)		10			10		7	3			10	
Kerry (S-OW-4)		10			10			10			10	

*T, Typical behavior; AT, Atypical behavior; ?, ambiguous behavior. The numbers indicate ratings of each rater's response choices in each situation*.

A Fleiss's kappa analysis (Fleiss et al., 2013; Gwet, 2014) was performed to determine whether there was agreement between the raters' judgment as to whether in each condition (choosing a seat, going through a door or a turnstile, navigating a public space, and trying on clothes) participants exhibited typical, non-typical, or not coded behavior. We followed guidelines from Altman (1991), and adapted those of Landis and Koch (1977), to interpret the level of agreement. The entire analysis is provided in Table 2. In the text, the status of each woman is indicated in brackets after her surname: NS prefix refers to women who did not have Surgery, and S Prefix refers to women who had surgery. NW was used for women with normal weight, OW for women with overweight status, and O1 or O3 for, respectively women with class 1 or 3 obesity. For women who had surgery, more information dealing with the number of months after surgery (3, 4, or 8 months) has been added. For example: Laura was a class 3 obesity woman who did not have surgery (NS-O3). Anita was a class 1 obesity woman who had surgery 3 months before she took part in the study (S-O1-3).

#### 2.3.1. Sitting on Public Transportation

There was a substantial agreement between the raters' judgments (κ = 0.72, *z* = 21.57, *p* < 0.001).

Results suggest that, while in contemporary France, in the Paris area, middle-class adults culture prefer to sit away from a neighbor when taking public transportation alone, but accept to be seated next to someone, and prefer to sit next to someone rather than stand, women with obesity do not follow this norm.

Our results show that women with normal weight (NS-NW), women with overweight status who never had surgery (NS-OW) and women with overweight status who had surgery a long time ago (S-OW-8) took a seat with a neighbor if that is all that was available.

**Figure 2:**
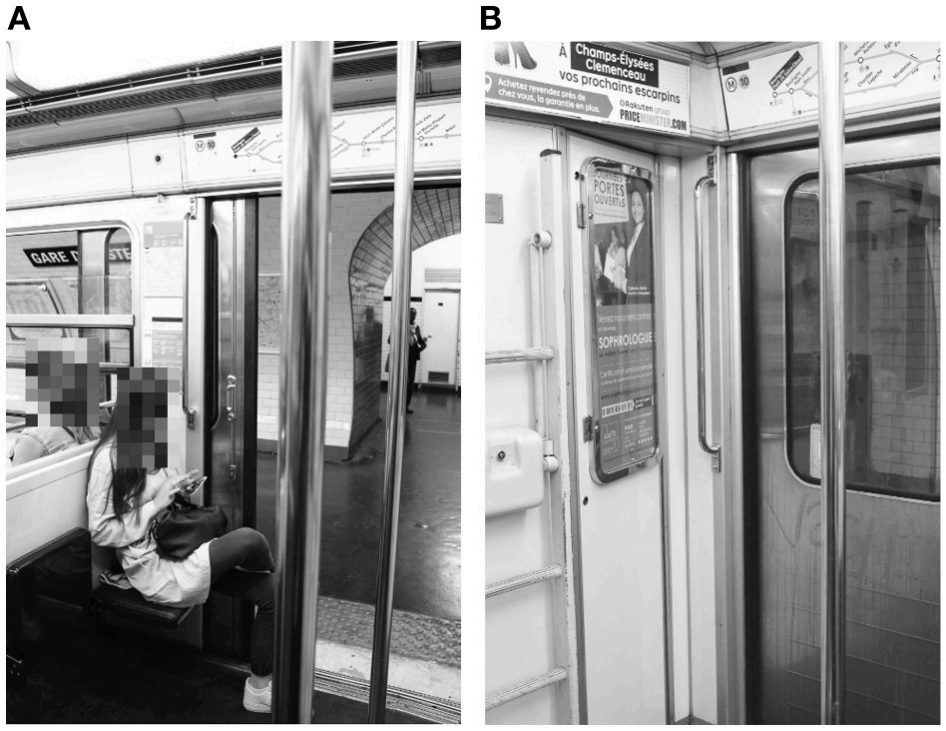
Failing to take a seat because there is someone already sitting in the next seat **(A)**. Deborah prefers to stand in the corner **(B)**. The participants to the study have given their agreement in writing for the publication of images from their subcam recordings, such as the above, provided they are not individually identifiable on the picture. Images have been blurred to ensure anonymity.

In contrast, all women with obesity (NS-O1, NS-O3, S-O1-3) and women with overweight status who had surgery (S-OW-4) avoided taking seats with a neighbor. They anticipated such situations and have built strategies to actively avoid finding themselves in the situation of sitting close to a neighbor on public transportation (see participants' comments in Appendix A for more details). Typically they rushed for the specific seats in train or bus that have no neighboring seat, or chose to stand. For example, Deborah (NS-O1) entered a subway car and spotted a free folding seat, but a woman was sitting in the next seat (Figure 2A); Deborah preferred to stand alone in the corner (Figure 2B).

#### 2.3.2. Going Through Turnstiles and Doors

There was an almost perfect agreement between the raters' judgments (κ = 0.88, *z* = 23.10, *p* < 0.001).

**Figure 3:**
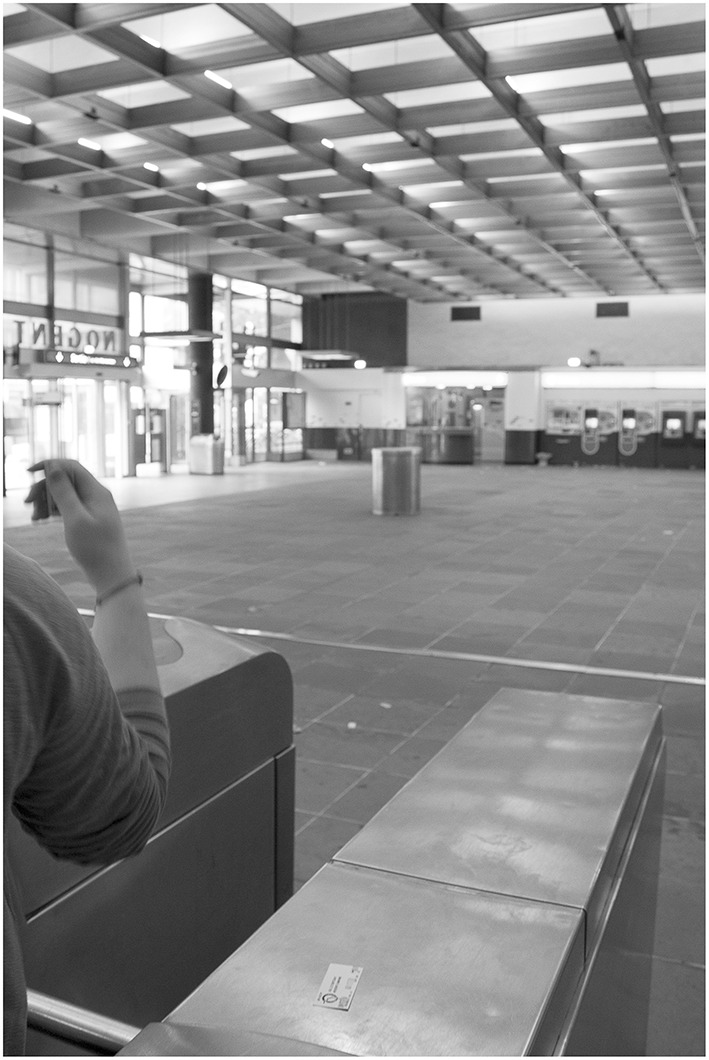
A woman with obesity passing sideways through the turnstile. The participants to the study have given their agreement in writing for the publication of images from their subcam recordings, such as the above, provided they are not individually identifiable on the picture. Images have been blurred to ensure anonymity.

In the French subway, there are doors for exiting the metro and turnstiles for entering it. It is interesting to see how participants deal with their body size in these two situations. Normal weight women, women with overweight status (NS-OW), or women with overweight status who had surgery long ago (S-OW-8) always went through doors and turnstiles frontways, as is typically expected. But we observed women with obesity (NS-O1, NS-O3) and recent ex-obese women (S-OW-4 and S-O1-3) going through the doors and turnstiles sideways (Figure 3; see Appendix B).

**Figure 4:**
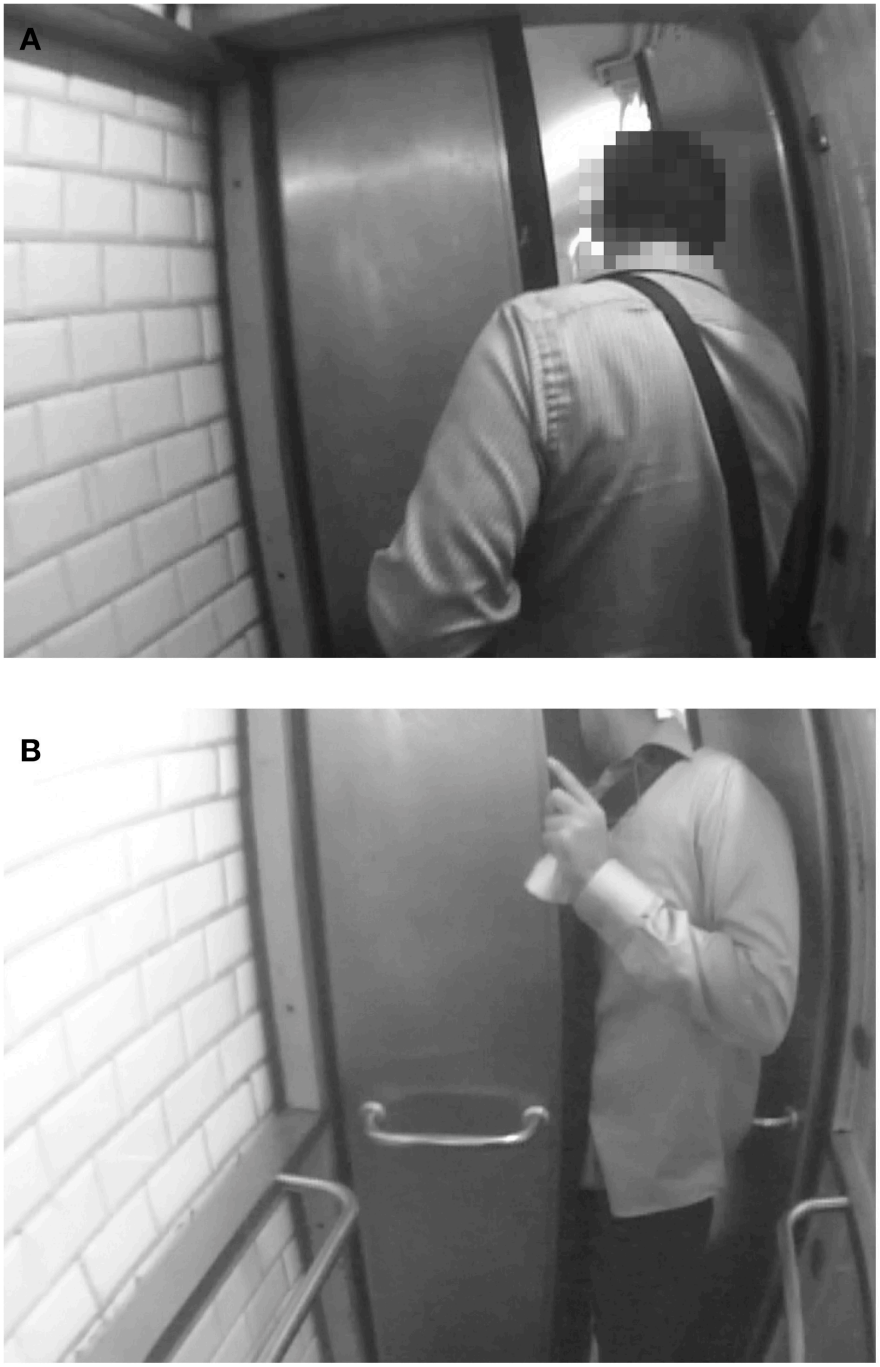
A man passing sideways through a blocked door **(A,B)**. The participants to the study have given their agreement in writing for the publication of images from their subcam recordings, such as the above, provided they are not individually identifiable on the picture. Images have been blurred to ensure anonymity.

The case of Deborah (NS-O1) is particularly illuminating as her behavior can be connected to an incident that happened the same day. When leaving the metro station earlier that day, Deborah had to go through a faulty exit door that was half blocked, leaving only a very narrow passage (Figures 4A,B). The figure shows how a man who went through the door just before her had to force his way through sideways, and Deborah did the same, with great difficulty. She did not enjoy this incident. She sounded quite angry and swore quietly for a few seconds afterwards, as we can hear on her recording. Then later the same day, she went through a large bank entrance door sideways, in a way typical of women with severe obesity. During the RIWs, Deborah said that this small humiliating episode involving the faulty exit door made her obesity very salient to her, thus contributing to her subsequent passing through the bank door sideways, which would only have been necessary if she had a far higher BMI. The daily occurrence of such embarrassing incidents reminding one of her large size is interesting to flag here for our analysis below.

#### 2.3.3. Navigating Public Spaces

There was a substantial agreement among the raters' judgments (κ = 0.63, *z* = 19.92, *p* < 0.001).

While women with normal weight (NS-NW) and women with overweight status (NS-OW) walked quickly in public space (street, metro corridors, shopping mall, etc.), sometimes passing other people, even at close range, and not hesitating to enter narrow gaps, all women with obesity (NS-O1, NS-O3, S-O1-3) tended to walk slower than the rest of the crowd. They avoided making sharp or fast maneuvers and generally navigated in a way that left ample space between them and obstacles or other people. Observed behavior of women with overweight status who had surgery (S-OW-4, S-OW-8) depended on the participant, being typical for some and atypical for others (see Appendix C). For example, Kerry (S-OW-4) showed atypical behavior, walking for 25 s behind a rather slow woman, before passing her although there was ample space on the right to pass her by. Viewers with normal weight (e.g., raters) mentioned they felt somewhat impatient while watching the tape. Kerry herself also mentioned in her RIW her slower and more cautious pace in navigation. However, this behavior was not systematic or constant among ex-obese women (who were overweight at the time of the study).

#### 2.3.4. Choosing Clothing Size

There was an almost perfect agreement among the raters' judgments (κ = 0.74, *z* = 22.34, *p* < 0.001).

One would expect participants to know their clothing size, at least approximately. However, during shopping, while participants with normal weight (NS-NW) selected clothes (coat or jacket) that fit their size, women with obesity (NS-O1, NS-O3, S-O1-3) and ex-obese women (S-OW-4, S-OW-8) tended to select clothes that are larger (see Appendix D).

Women with overweight status and women with obesity have problems with selecting the correct size and tend to overestimate their measurements and/or take larger sizes than they need to avoid wearing something tight. They take some garments into the fitting room, but when they try them on, they realize these clothes are much too big for them. For example, Karen (S-OW-4) tried on a coat in a shop and looked in the fitting room mirror. She found that she was thinner than she thought. She then looked into another large mirror at the other end of the fitting room, then walked up to a girl sitting there and said, “Can I ask you something? In your opinion, does this mirror make me look thinner?” The girl looked at the mirror and replied, “Er, uh, I don't know,” and then asked another girl in the fitting room, “Hey, do you think the mirror makes you look thinner?” The other girl replied (popping her head out of her fitting room first in the mirror, then at Karen “No (to Karen), it's normal.” In our sample everyone except women with normal weight experienced issues with size.

### 2.4. Discussion

Our observations showed that women with obesity displayed several atypical behaviors. These observations were confirmed by the interviews, where participants acknowledge and explain, as shown in Appendices. (1) Women with obesity actively searched for seats where they would not risk encroaching on their neighbor. They used specific strategies for this. (2) They tended to go through doors and other narrow passages (such as metro turnstiles) sideways, even when they could go through frontways. (3) They navigated the environment more cautiously and slowly: they did not take sharp turns, avoided entering narrow spaces, gave way or left more space when passing other people, hesitated to pass slow people and were themselves passed by most other pedestrians, and rarely moved swiftly and boldly when obstacles were close by or when other people were moving quickly. (4) They had issues with knowing their correct clothing size and tended to overestimate it. The higher the BMI, and the higher the body perception index (BPI), the more these behaviors were salient.

In others groups, behaviors varied depending on the situation. With regard to passing through turnstiles, doors, and narrow passageways sideways, women with obesity and ex-obese women who had recently had surgery (and were overweight at the time of the study) tended to behave atypically, while those who had surgery a long time ago behaved more like normal weight or overweight women. With regard to navigating public spaces, only women with normal weight and two ex-obese women (who had surgery and were overweight at time of the study) moved swiftly. Other groups behaved like women with obesity. Regarding clothing, all but women with normal weight seemed to have difficulties selecting the right size.

Furthermore, it seemed that in the various situations studied, it took time for women with obesity who had surgery to abandon their previous behaviors: only those who had surgery a long time ago (8 months) behaved like normal weight women when sitting on public transportation or going through doors and turnstiles, but they still had difficulty in choosing the right clothing size or, for some of them, navigating public spaces.

These data raise two issues. The first is that obesity, apart from its medical implications, is also a challenge in performing mundane activities because the built environment, designed for normal weight persons, presents challenges for women with obesity. Regarding seats, the problems are obvious. Regarding clothing, that is a classic issue (larger sizes are often mostly a linear extrapolation of smaller sizes, while shapes do not change linearly). Finally, regarding doors, turnstiles, and pedestrian traffic, as we have seen above, they can also be an issue, which is less well-known. In all these situations, women with obesity are reminded, in a negative way, of their body size, and this is likely to create, sustain, or enhance stigma.

Regarding stigma, the RIWs clearly showed that body image issues evoke the social image (looks from others), which is perceived as negative (see Appendices). Women with obesity, and to some extent women with overweight status, express the feeling that they are disturbing and cumbersome, take up too much space, do not look good, and are a source of annoyance for others. Discussion of their behavior often referred back to memories of humiliating past experiences. On several occasions, participants mentioned that awareness of their body image was a source of concern continuously present in their mind, at least in some situations. All the above suggests that body image is a matter of concern for women with obesity and overweight status and that mundane activity makes this concern salient quite often (likely, several times a day for those who travel on public transportation).

Another issue arises from our observations that the behavior and experience of participants who have a larger-than-standard body size seem to be *disproportionate* to their actual affordances. This is evident, for example, when going through large doors sideways, navigating more cautiously than the rest of the crowd, and exhibiting concern about encroaching on people sitting next to them more than other passengers, as in the example of Dorothy. In other words, it seems women with obesity (and to a lesser degree women with overweight status) behave as if their body was even larger than it is. The fact that ex-obese women continue, for at least a few months after surgery, to act as if they were individuals with obesity supports this hypothesis. We are not the first to report such finding. For example, Cash (2004) studied how individuals who were currently or formerly with overweight, and individual who were never overweight differ in a range of eating and body image measures. The author reported residual body image problems following weight loss. When they lose weight, people with overweight status in the past do not ultimately obtain the same positive body image as someone who has never been overweight.

This finding supports the idea that body image is an internal construct of a unitary corporeal self that endures in space and time, and it seems that, in the post-surgery period, the representation has more inertia than the body itself. In other words, the represented body image would differ from the perceived body image, that is the image that is objectively perceived by the person (such as in looking at oneself in a mirror). In our case indeed, it seems that the represented image of women with obesity was larger than the perceived image since they behaved as if their bodies were larger than it actually was. This will be one of the hypotheses investigated in study 2. It is also interesting to measure the overestimation, and to see if it varies for different classes of BMI. This will also be investigated in study 2.

## 3. Study 2: Body Size Among Women

Study 1 showed behaviors and their rationale as described by the women with obesity, as well as the way they relate them to embarrassing past social experience, suggest (a) that women with large body-size actively avoid situations where their size would expose them to “embarrassing” situations where they would appear to be in the way of others and (b) as these precautions are excessive compared to actual affordances, that they tend to overestimate their size, which is confirmed for instance by their overestimation of clothing size.

Study 2 explores how actual body-size impacts the representations of the body: is there actually an overestimation of body size by larger women, and is this is a matter of perception or representation?

The literature on body size in women with obesity is difficult to summarize, as different studies support three very different conclusions: the women overestimate, underestimate, and are accurate regarding body size estimation (Schwartz and Brownell, 2004). The inconsistent findings across this literature are potentially due to different methods of measurement and samples that vary in crucial aspects (Mills and Fuller-Tyszkiewicz, 2016; Castro et al., 2017).

Studies differ with regard to methods of assessment (Johnstone et al., 2008): while some studies used traditional figure rating scales, others used the more advanced whole-image adjustment procedures including photo distortion or morphing (Farrell et al., 2003; Urdapilleta et al., 2007), which is the method we use in this second study, in which we investigate the different dimensions of body image.

This distinction between the components of body image is interesting because it may help us to better understand the nature of body size estimation. Authors (Farrell et al., 2003; Docteur et al., 2012) make a difference between body image perception (i.e., perceived body size, as seen in a mirror) and body image representation (i.e., recall body size). To measure body image perception, participants are asked to adjust their modified photograph to match their image in a large traditional mirror, using direct visual information (“perception condition”). For body image representation, participants are asked to adjust their modified photograph in the absence of a photo or mirror at the time of testing. Participants then have to estimate their size from their own memory (“representation condition”).

Mirrors allow us to view our own body from a third-person (observer) perspective. However, as mentioned by Preston et al. (2015), how viewing ourselves through a mirror affects central body perception compared with a true third-person perspective is not fully understood.

Moyer et al. (1978) first provided support for the idea that size estimations differ for perceived and remembered sizes and found that estimations from memory tend to be larger than estimations from perception of objects. This finding was replicated in a study by Farrell et al. (2003), within the specific context of body image estimation. In contrast, by comparing body perception and body representation in 55 women with normal weight, Farrell et al. (2003) found the opposite effect, namely, judgments made from perception tended to be larger than those made from memory, but in that case more accurate. The authors noted, “The finding that participants were more accurate in estimating their body size with a mirror in front of them than without is counterintuitive” (p. 169). The same task was performed in a more recent study with 91 women with normal weight (Docteur et al., 2012). Results showed that participants were accurate in the mirror condition (with only 1.15% overestimation for body perception with a mirror) but less accurate in the second condition (5.25% overestimation for body representation, with no mirror).

To our knowledge, only a few earlier studies have used the morphing technique with a mirror to investigate persons with obesity (Shipman and Sohlkhah, 1967; Gardner et al., 1989). Shipman and Sohlkhah (1967) showed that persons with obesity were less accurate in estimating their body size than persons with normal weight, but Gardner et al. (1989) found that even though participants were more accurate with a mirror, there were no significant differences between persons with obesity and persons with normal weight.

Therefore, more data on persons with obesity are needed, as understanding body image and its consequences for a person's life is a key aspect of the issue of behaviors related to body size in obesity. As our Study 1 showed, it seems that the represented image (how people imagine they are) is larger than the perceived image (how people perceive themselves) for women with obesity and overweight status, because they behaved as if their body were larger than it actually was. However, the population concerned by this overestimation process remains undefined. This process could concern all women or only women with a large BMI.

Thus, we hypothesized that (1) all women would overestimate their body size representation (recall condition) more than their body size perception (mirror condition). (2) for all women, the higher their BMI, the more they would overestimate their body size (in perceived and recall conditions). Finally, we hypothesized that (3) the higher the BMI, the greater the difference between body size in perceived and recall conditions.

Because women in our study who had surgery quickly lost weight in a few months, we expected that evaluating body size would be more difficult for them than for women who did not lose weight, because it takes time after weight loss to get used to one's new body and accurately estimate one's body weight. So, we hypothesized (4) that women who had surgery would more overestimate both their perceived and recall body size than women who never had surgery and (5) this overestimation of both perceived and recalled body size according to BMI will be higher for women who had surgery than for women who had never had surgery. Finally, we hypothesized that (6) the difference between body size in perceived and recall conditions will be higher for women who had surgery than for women who had not surgery.

### 3.1. Materials and Methods

One hundred and forty French women, aged 20–45 (*M* = 27.36, *SD* = 5.51), took part in this study. Participants included women with different BMI (as in Study 1). Some of them never had surgery (*n* = 80) and others had bariatric surgery (*n* = 60). See Table 3 for participants' characteristics.

**Table 3:** Body Size Index (BSI) in the Recall (R) and the Mirror (M) conditions and BMI for all groups of participants (mean and standard deviation).

	**No surgery**				**Surgery**		
	**Normal weight (NS-NW)**	**Overweight (NS-OW)**	**Obesity class 1 (NS-O1)**	**Obesity class 3 (NS-O3)**	**Overweight 8 months after surgery (S-OW-8)**	**Overweight 4 months after surgery (S-OW-4)**	**Obesity class 1 3 months after surgery (S-O1-3)**
BSI-R	22.30^a^ (2.17)	29.21^b^ (2.36)	42.34 (2.96)	59.25 (4.04)	34.33 (1.95)	37.62 (2.41)	49.70 (4.05)
BSI-M	22.36^a^ (1,92)	28.99^b^^,^^c^^,^^d^ (2.42)	35.62 (2.41)	48.17 (3.22)	28.91^d^^,^^e^ (1.71)	31.61^c^^,^^e^ (2.11)	41.27 (3.40)
BMI	21.11 (1.22)	27.05 (1.60)	32.32 (1.62)	42.58 (2.36)	26.42 (1.00)	28.65 (1.40)	34.18 (0.48)

*All differences between cells are significant according to Tukey post-hoc tests (all ps <0.05) with the exception of those indicated in notes below. Only Tukey post-hoc tests comparing groups one by one (BSI-R and BSI-M for Normal Weight, for example) and comparing all groups for only BSI-R or BSI-M are reported in notes below*.

a *t_(132)_ = 0.09, SE = 0.56, p = 1.00*.

b *t_(132)_ = 0.40, SE = 0.54, p = 1.00*.

c *t_(192.32)_ = -2.99, SE = 0.87, p = 0.15*.

d *t_(192.32)_ = 0.09, SE = 0.87, p = 1.00*.

e *t_(192.32)_ = 3.08, SE = 0.87, p = 0.12*.

Participants' education level ranged from BTEC First Diploma to Masters' degree. Written informed consent was obtained from each participant and the research followed all applicable institutional and governmental regulations concerning the ethical use of human volunteers. The protocol was validated by the Social Psychology Ethics Committee at the London School of Economics and Political Science (Houghton St, WC2A 2AE, London, UK). All participants were volunteers and were free to withdraw at any stage of the study. No remuneration was given. Finally, all participants were given a special telephone hotline in case they had second thoughts or questions. Written informed consent was signed by the participants.

Women were recruited through the hospital where they had or will have surgery, through a call for volunteers, and through snowball sampling, starting with a convenience subsample of university employees.

Each participant was tested individually. First, a woman experimenter took a digital photograph of the participant in street clothes (jeans and T-shirt) in front of a white wall. Then the resulting photograph was randomly enlarged or slimmed down (+25 or −25%) using the previously validated computer program Anamorphic Micro^©^ Software (Urdapilleta et al., 2007, 2010; Docteur et al., 2010). Then, the woman experimenter showed this enlarged or slimmed photographs to the participant, who was asked to modify her enlarged or slimmed photographs onto the computer by sliding a cursor “until the photograph matched her current size.” Participants agreed in writing that their photos be processed by computer morphing software image and their photographs be used in the experimental framework of this study, and be the object of communications and publications to the extent that their face will be blurred.

However, the last step of the study (matching the photo) differed as a function of the experimental condition. Half of the participants (condition 1: recall) were asked to adjust their modified photograph in the absence of a photo or mirror at the time of testing. Therefore, participants were asked to rely on their own memory as a reference when adjusting their modified image to match their size. The mental representation served as a basis for comparison since there were no other cues: body size *representation* (how people represented, imagine, how they are) was therefore measured. In condition 2 (mirror), participants were asked to adjust their modified photograph to match their image using existing visual information: a large, full-size classic mirror was provided next to the computer. In the second condition therefore, as participants adjusted their modified image when standing in front of this classic mirror, body size *perception* (how people perceive themselves) was measured.

All participants completed the recall task first, then the mirror task. At the end of the session, each participant's actual weight and height were measured and were used to calculate their BMI. In the present study, the BMI has been considered as an independent variable, in accordance to the recommendations proposed by Smeets et al. (1998). The software provided an estimation score (ES) by comparing the individual's response (the Estimated Size, which is the image as adjusted by the subject) to the actual image. ES = [Estimated Size (in pixels) / True Image Size (in pixels)] × 100. For example, an ES = 101.15 corresponds to a 1.15% overestimation.

**Figure 5:**
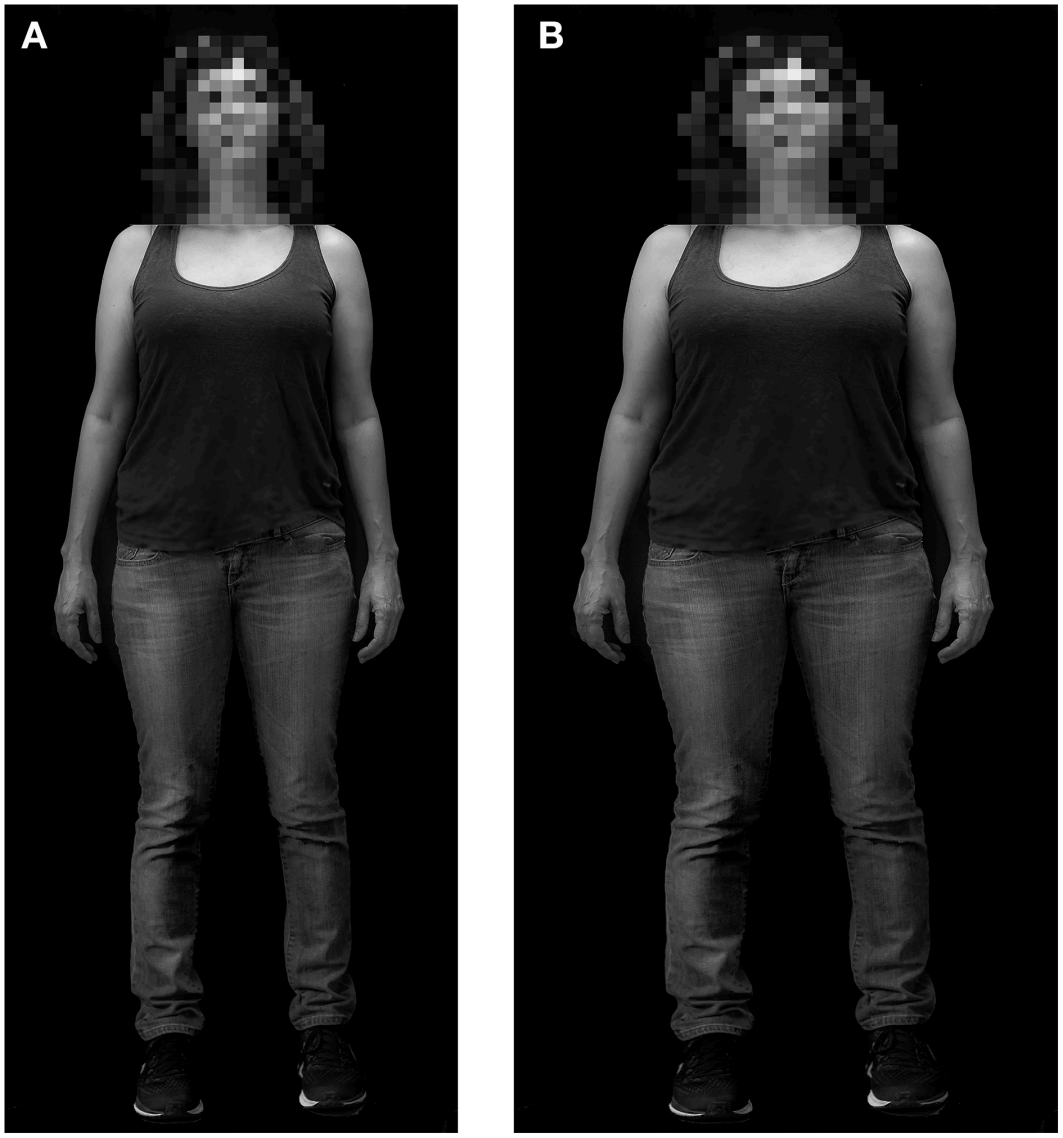
Example output from the photographic morphing procedure **(A,B)**. Participant agreed in writing that their photos be processed by computer morphing software image; and their photographs be used in the experimental framework of this study, and be the object of communications and publications to the extent that their face will be blurred. **(A)** Real photograph of the participant; i.e., the actual body size of the participant (BMI = 21.08). **(B)** Photograph modified by the participant; i.e., the estimated size as adjusted by the participant. In this case, the estimation score is 120%, or 20% larger than the actual size. The BPI is 25.30 (21.08 × 120%).

The method described by Farrell et al. (2003) was used to analyze these data. These authors calculated a Body Perception Index (BPI) based on the following index: BPI = BMI × ES, where BMI is the Body Mass Index of the participant and ES the Estimation Score defined above. Estimation score is measured in m^2^/m^2^, and BPI in kg/m^2^ × (m^2^/m^2^) = kg/m^2^ (for a detailed discussion of this index, see Smeets et al., 1998). BPI is an indication of participants' subjective perception of their BMI. The BPI is the BMI that would correspond to the estimate given by the participant. Using the BPI is interesting because it provides a way of comparing the response of the participant to a social norm. For example, if a participant has a BPI over 30, one can say that her representation of herself would fall within the obese category, based on socially accepted criteria. Figures 5A,B show examples from the photographic manipulation software. Because we need to measure an index for body perception and one for body representation, we used two indexes: BSI-M (how people perceive themselves; i.e., body size perception, BPI calculated in the Mirror condition) and BSI-R (how people imagine they are; i.e., body size representation, BPI calculated in the Recall condition).

BSI as a dependent variable was analyzed using the R software following mixed linear model procedures with random slopes (Pinheiro and Bates, 2000; Bates, 2005). Analyses were performed with the *nlme* package computed by Pinheiro and Bates (2000) in R (3.2). Degrees of freedom were calculated according to Pinheiro and Bates (2000). Conditions was a two levels within factor (recall and mirror), group was a two levels between factor (Bariatric Surgery and No Bariatric Surgery), and BMI was a continuous factor. Results were considered to be significant if *p* < 0.05.

### 3.2. Results

The first set of hypotheses concerned the effect of conditions. The analyses revealed a main effect of the condition. In the recall condition (BSI-R) the mean was significantly higher than in the mirror condition (BSI-M), with β = 12.33 (*SE* = 1.18), 95% CI [10.00, 14.66], *F*_(1, 136)_ = 109.51, η^2^ = 0.45, *p* < 0.001. In fact, in accordance with our second hypothesis, the BSI (BSI-R and BSI-M) increased as a function of BMI, with β = 1.75 (*SE* = 0.031), 95% CI [1.69, 1.81], *F*_(1, 136)_ = 3092.91, η^2^ = 0.96, *p* < 0.001. Finally, concerning the third hypothesis, the higher the BMI, the greater the difference between BSI-R and BSI-M, with β = -0.55 (*SE* = 0.04), 95% CI [−0.62, −0.47], *F*_(1, 136)_ = 217.74, η^2^ = 0.62, *p* < 0.001.

The second set of hypotheses concerned the effect of surgery. Contrary to the fourth hypothesis, a non-significant difference was found concerning accuracy in estimating body size (BSI) between women who had surgery and women who had not, with β = −2.69 (*SE* = 2.77), 95% CI [−8.16, 2.78], *F*_(1, 136)_ < 1, η^2^ = 0.01, *ns*. In concordance with the fifth hypothesis, the higher their BMI, the more women overestimate their body size (BSI-R and BSI-M), and this overestimate tends to be higher for women who had surgery than for women who had not, with β = 0.23 (*SE* = 0.09), 95% CI [0.05, 0.41], *F*_(1, 136)_ = 6.12, η^2^ = 0.04, *p* = 0.015. Finally, the difference between BSI-M and BSI-R was higher for women who had surgery than for women who had not, with β = −7.15 (*SE* = 3.25), 95% CI [−13.58, −0.73], *F*_(1, 136)_ = 4.85, η^2^ = 0.03, *p* = 0.029 (see Table 3 for means and standard deviations).

Tukey *post-hoc* tests were run on the previously studied groups. Linear models allow to studying variables and their interactions. However, for this study to be complete, one should also focus on the comparison between BPIs for the groups examined in Study 1. Results are shown in Table 3.

One should also note that the overestimation for women who had surgery was between 28.85 and 35.35% in the recall condition. For other women with obesity who had not surgery, the overestimation was between 29.75 and 39.30%. Only women with normal weight (5.95%) and women with overweight status (7.95%) present a low overestimation. Results are presented in Table 4.

**Table 4:** Estimation Score (ES) in percent (%) in the Recall (R) and Mirror (M) Conditions for the all groups of participants (mean and standard deviation).

	**No surgery**				**Surgery**		
	**Normal weight (NS-NW)**	**Overweight (NS-OW)**	**Obesity class 1 (NS-O1)**	**Obesity class 3 (NS-O3)**	**Overweight 8 months after surgery (S-OW-8)**	**Overweight 4 months after surgery (S-OW-4)**	**Obesity class 1 3 months after surgery (S-O1-3)**
EI-R	5.95^a^ (2.17)	7.95^b^ (2.36)	29.75 (2.96)	39.30 (4.04)	28.85 (1.95)	30.40 (2.41)	35.35 (4.05)
EI-M	5.05^a^ (1.92)	6.10^b^ (2.42)	9.10 (2.41)	13.45 (3.22)	9.40 (1.71)	9.35 (2.11)	11.10 (3.40)

*All differences between cells are significant according to Tukey post-hoc tests (all ps <0.05) with the exception of those indicated in notes below. Only Tukey post-hoc tests comparing groups one by one (EI-R and EI-M for Normal Weight, for example) are reported in notes below*.

a *t_(133)_ = 0.65, SE = 1.39, p = 1.00*.

b *t_(133)_ = 1.33, SE = 1.39, p = 0.99*.

### 3.3. Discussion

The aim of this second study was to answer the following questions. How do women with different BMI, women with obesity and ex-obese women, who had or not bariatric surgery, view their body size? Would the represented body image differ from the perceived body image? This aims at understanding why women with obesity behave as if they had a larger body than they actually have (results of study 1). Is that because they perceive (through their senses) their body size larger than real, or because they represent (in their mind's eye, i.e., recall) their body as larger than real? This matters because representation is socially constructed and involves social judgment. Mead (1972), in personality construction, makes the distinction between the *I* (the subject who acts) and the *Me*. Me is the image of self internalized based on experience of interaction with others, which in the case of persons with obesity might involve social stigma. The recall condition here elicits the “Me” aspect of self.

To answer these questions, body size in a mirror condition was measured to investigate how women saw themselves (perception) and body size in a recall condition to investigate how women imagined themselves to be (representation). Women whose BMI varied from normal weight to obese class 3 (some of them had bariatric surgery and others never did) were asked to adjust a (modified) photo of themselves to match their actual size.

Results concerning the first set of hypotheses showed that participants perceived their body size as being larger in the recall than in the mirror condition (as predicted in the first hypothesis). Tukey *post-hoc* tests shed some light on these results and, in fact, no differences were observed between women with normal-weight (NS-NW) and women with overweight status who had no surgery (NS-OW), in regard to perception of their body size according to the two conditions.

The larger participants actually were (with higher BMI), the more they seem to overestimate their size in both conditions (mirror and recall), as predicted in the second hypothesis. Furthermore, the more corpulent women are, the greater the difference between representation and perception of their bodies (representation being larger than perception).

One should note that the accuracy of size estimations was quite good for all groups in the mirror condition (about 5–13% in the mirror condition). In the recall condition, for women with normal weight (NS-NW) and women with overweight status with no surgery (NS-OW), the error of size estimation was about 6–8%, but it was about 30–40% for all women with obesity (NS-O1, NS-O3, S-O1-3) and women with overweight status at 4 and 8 months after surgery (S-OW-4, S-OW-8).

Our results support those of a previous study (Docteur et al., 2012) comparing body size estimation in the presence or absence of a mirror, in which the presence of a mirror makes the estimations more accurate. It can be argued that seeing one's image in a mirror and then adjusting one's photograph on a computer does not rely on the memory of one's own life-size image but is rather a stimulus-matching task. In contrast, a recall estimation based on representation involves a memory judgment rather than visual information, and includes cognitive, attitudinal, and affective components (Thompson, 1996) and feelings concerning one's own body (Cash, 2004), which may affect body size estimation (Smeets and Panhuysen, 1995).

Our results also support other previous studies on the effect of one's personal body size on the accuracy of estimating their own body size. Thaler et al. (2018) tested whether one's personal body size predicts the accuracy of body size estimation of own body size. Fifty-four women were presented with their personalized avatars varying in weight in a virtual environment and responded whether the body presented corresponded to their actual body size and adjusted the avatar until it matched the size they perceived their actual body to be. Results show that participants' BMI significantly altered the accuracy of estimated own body size; participants in the overweight status and obese weight range tended to overestimate their body size, but participants with lower BMI underestimated their body size.

Contrary to the fourth hypothesis, a non-significant difference was found concerning the overestimation of both perceived and represented body size between women who had surgery and women who did not had surgery. In concordance with the fifth hypothesis, the overestimation of BSI in both experimental conditions tends to be higher for women who had surgery than for women who did not have surgery. Thus, differences between women can be revealed only if their actual body size (BMI) is considered, which is in line with the recommendation proposed by Smeets et al. (1998). The overestimation in the recall condition compared to the mirror condition was also higher for women who had surgery than for women who never had surgery (the sixth hypothesis).

Finally, unplanned comparisons (Tukey *post-hoc* tests) between groups for the recall condition revealed significant differences. This set of results means that overweight women who never had surgery (NS-OW) had a better accuracy in estimating their body size than women with overweight status at 8 or 4 months after surgery (S-OW-4, S-OW-8). Moreover, women with obesity class 1 (NS-O1) who did not had surgery had better accuracy in estimating their body size than women with obesity 3 months after surgery (S-O1-3). In fact, it seems that women who had bariatric surgery, even when they lost weight and became women with overweight status or obesity class 1, displayed levels of overestimation of their body size. This could be explained by the fact that they do not have the same perception of their body size as women with the same BMI, who were not women with obesity class 3 in the past, before surgery.

It seems that it takes time after surgery to achieve a non-erroneous perception of one's body size. One should consider that the weight of women who had surgery might fluctuate more than the weight of participants with normal weight.

## 4. General Discussion

In this paper, two studies were presented. In the first, we investigated how women with obesity act with their body in natural situations. We analyzed the activity of women with obesity and ex-obese women who had lost weight after surgery (we also compared it to that of women with overweight status and normal weight). In the second, using an experimental protocol, we explored the body size representation and perception of French women. Results showed that women with obesity in our sample do behave differently from women with normal weight or overweight status in certain circumstances: they tend to avoid sitting next to other people, go through doors and turnstiles sideways, navigate more carefully in a crowded space, and experience difficulties in selecting the right size of clothing. These behaviors are not systematic, but frequent, and the higher the BMI, the more salient they are. Interestingly, ex-obese women who have recently lost weight tend to continue to behave as if they were women with obesity. However, these specificities seem to vanish with time. It seems that women with obesity, and to a certain extent women with overweight status, behave as if their body were larger than it really is.

Note that other studies including women with low BMI have found similar effects. For example, Guardia et al. (2012a) using an ecological paradigm (Guardia et al., 2010) in which anorexic women required to judge whether or not an aperture was wide enough for them to pass through, show that they significantly overestimated their own passability (relative to a control group) in a simulated body-scaled action. This body overestimation appears to be related not only to the anorexic women's body image but also to an abnormal representation of the body in action. With anorexic women the body-boundary and the body-orientation representation seem disturbed (Guardia et al., 2012b).

The second study showed that women with obesity and women with overweight status who had surgery overestimated their size when estimations were based on representation (recall condition, without mirror) by around 30–40% but overestimated much less in the mirror condition. This supports the hypothesis that the represented size (rather than the actual size) is the operational body size for behavior and activity. While one could expect that motor behavior would rely on perception (and proprioception) rather than representation, this does not seem to be the case for these women: their behavior seems to correspond to their oversized representation rather than to their body shape perception. This suggests that a possible interpretation of the atypical behaviors observed empirically in persons with obesity is not (or not only) due to the use of perceived size, such as the measure of affordances, but rather due to an excessive margin of behavioral precautions to avoid situations which may be humiliating and stigmatizing (Brewis et al., 2011; Major et al., 2014). In other words, persons with obesity may have larger personal space boundaries than persons with normal weight or overweight; and this would be reflected in the way they move in public spaces. Persons with obesity would maintain a greater distance in order to keep a safe distance and avoid contact.

What seems specific is that this fear of getting too close is not a fear of being touched but rather a fear of intruding into other people's personal sphere and being a nuisance (see comments of participants in Appendices). What makes the situations unpleasant would be therefore not only the feeling of being “rubbed against” or squashed, but also the feeling of being stared at by others. Nevertheless, an analysis of the replay interviews suggests that concluding that the only mechanism producing this behavior is that the represented size is the operational size may be too simplistic. Rather, while our experiments are able to attribute a size to the represented body, body image is more than a size, and only by also listening to the interviews can we get a glimpse of what the represented body size means. It comes with negative connotations. RIWs show that the atypical behaviors are connected to memories of embarrassing or humiliating experiences regarding personal body size (see Appendices). Participants explicitly said that avoiding repeating such unpleasant experiences is the rationale behind some of the atypical behaviors. This is quite obvious in some of the verbatim quotes provided in Appendices regarding seating (e.g., *encroaching* on a neighbor's space). The presence of such key autobiographical events related to the experience of the body confirms the presence of episodic memory in the representation of the body as proposed in the body matrix model (Riva, 2018). It is interesting to note here that these biographic elements which are very social in nature are evoked by a first person-video, showing an integration of allocentric and egocentric frames that is reinforced by the stigma. In this respect the stigma acts as a factor of integration of negative body image, through negative emotional experiences.

However, this goes beyond specific situations: if we turn to the replay interviews, it appears that obesity (whether present, or past) is connected with shame and guilt, and can be linked to memories of unpleasant experiences (see the RIWs in Appendices). Some women with obesity commented that they felt anxious in daily interactions with other people. The example of Deborah (NS-O1) with the blocked door described above suggests that the fear of experiencing a size-related unpleasant or humiliating experience could be triggered or reactivated and made salient by some incident that made obesity more salient (e.g., rubbing against door frames, encroaching on neighbors' space, blocking the way etc.). We can assume that women with obesity statistically encounter enough of such reactivating experiences to keep them continuously aware and on guard against such situations. Therefore, women with obesity may actively try to avoid situations in which they may be pointed at or humiliated again. That is why they declare being afraid of overloading lifts, of encroaching on neighbors' space on chairs, and of disturbing people by taking up too much space in public areas.

Exaggerated representations could be considered as having an adaptive value. There is a social psychological cost in making a mistake, and persons with obesity, by extending may extend the safety distance, avoid embarrassment. One may think that persons with obesity are simply using the standard representations of how to use space, and how one should use space, but they use them with an “incorrect” (oversized) assumption of their own body size (they overestimate their body size by about 30%). The result is quite coherent: they would avoid doing a series of things which would indeed have a negative consequence (rubbing against doorposts, encroaching on neighbors' space, blocking the way etc.) if they were as large as they thought they were. Socially, they would feel the need to apologize for the inconvenience that they (think they) represent. Therefore, they would feel they are a special case who obstructs the swift flow of normal activity (slowing traffic in corridors), restrict other people's space (in public transportation or space), and are a danger to furniture (chairs, wheelchairs, etc.) In addition, because obesity is considered to be the result of one's own failings (greediness, laziness, a sign of excess and lack of control), persons with obesity feel they are in the position of someone who is at fault, and act accordingly (Lee and Pausé, 2016; Seacat et al., 2016; Flint et al., 2017).

All this means that persons with obesity may feel they have (in representation) violated social or moral rules, and therefore feel they are guilty and should take a low profile and/or apologize, which is what can be observed. Not only is there a stigma attached to obesity, as there is to many appearances or behaviors that deviate from the norm, but this stigma is probably proportionate to the degree of deviation from the norm (which is consistent with our finding that overestimation grows with BMI). The fact that persons with obesity overestimate their difference creates anticipations of strong stigmas and keeps them on their toes; this increases the stigma. The causality may go both ways: own-body size is likely to be overestimated precisely because obesity is a stigma; but then this overestimation increases the stigma.

In the perspective of integrative models of the body mentioned in discussion, our empirical findings suggest that stigma plays a role in integrating the (egocentric) emotional and kinesthetic experience and the (allocentric social) frames of reference in what seems a self-vicious circle, while the environmental affordances (e.g., small chairs, narrow turnstiles) provide on a daily basis a reinforcement of negative experiences that feeds this circle. This suggests that using virtual reality (Riva, 2011; Serino et al., 2016) is indeed a fruitful avenue that could compensate reinforcement by the usual environment of the subjects.

Early studies have shown that the mental size of an object can be influenced by its relevance to the viewer. For example, children tend to overestimate the size of coins compared to the size of paper discs of identical diameter (Bruner and Goodman, 1947). Authors referred to this as “accentuation,” a central process which leads to systematic tendencies in attributive judgment, increased saliency of the personally relevant (Bruner and Postman, 1949). While this notion did not have a strong follow-up, it seems relevant in our case, where persons with obesity appear to have a heightened sensitivity to what is relevant to their obesity (for example, the looks that other people give them, or their opinions). It is as if there had been some hypersensitization to the issue. The problem is that while these behaviors are excessive, they are not completely unfounded. For example, it is indeed more difficult to navigate with a larger and heavier body; persons with obesity do encroach on the next seat when they sit in narrow seats, etc. Persons with high BMI are aware of this and, as we saw, are oversensitive to these issues. In fact, in interviews women with obesity sometimes explicitly expressed a surprisingly harsh evaluation of themselves, certainly far harsher than persons with normal weight would venture to express. It seems that the issue is simply that the problems, although real, are overestimated. In a way, what is observed is similar to what was evidenced by the photo morphing experiment: persons with obesity do not have an imaginary problem, but they exaggerate its extent, relative to objective affordances and probably to social relations. This accentuation makes their life even more difficult.

### 4.1. Limitations

However, there are several limitations to our research. Firstly, this study is limited to French women in urban context. Still, body image literature reviews have revealed significant ethnic differences (Dorsey et al., 2009; Hebl et al., 2009). For example, Gramaglia et al. (2018) showed that Japanese women's ideal BMI and body shape are, respectively, lower and thinner than that of American women; or that Hispanic and Black women usually show less anti-fat attitudes than White women. It also seems that in some cultures obesity does not come with the same type of stigma (Hebl and Heatherton, 1998; Greenleaf et al., 2006). As this study is limited to French women of Urban culture, it would be interesting to survey other populations.

We know from pilot studies that men have a somewhat different relation to obesity even if a significant number of men do struggle with body image concerns (Pope et al., 2000; Ricciardelli et al., 2007). They also seem engaged in negative body talk (Engeln et al., 2013) and suffer of weight stigma (Himmelstein et al., 2018). Future research should attempt to study men's behavior and the role of gender or gender socialization on behavior in public space.

Secondly, we must note, on the one hand, that participation was made on a voluntary basis, and therefore there might be a self-selection bias in the sample. Another limitation of the present study relates to the small sample size for study 1 (*N* = 14), even if ethnographic studies often rely on much smaller numbers sample sizes. Replication of these results in a larger sample is desirable. It would have been interesting to include participants who had bariatric surgery very long time ago and managed to keep normal weight for a long time, but such participants are rare and difficult to reach.

Finally, future research should attempt to determine the potential variables associated with obesity-related behavior in daily life and body image problems in persons with obesity. It would be especially relevant to measure the emotional component of body image to assess emotional states associated to the perception of self-images by women. It might enlighten how the various components of body representation affect behavior.

## Data Availability

For study 1, the datasets for this manuscript are not publicly available, because the images have been blurred to ensure anonymity, but not the films used. For study 2, raw data supporting the conclusions of this manuscript will be made available by the authors, without undue reservation, to any qualified researcher.

## Author Contributions

IU and SL contributed to the conception and design of the experiments, acquisition of data, analysis and interpretation of data, and drafting of the article. SD contributed to the analysis and interpretation of data, and drafting of the article. J-MC contributed to the conception and design of the experiments.

### Conflict of Interest Statement

The authors declare that the research was conducted in the absence of any commercial or financial relationships that could be construed as a potential conflict of interest.
