# Supplementary material for: Women With Obesity Are Not as Curvy as They Think: Consequences on Their Everyday Life Behavior
Source: Front Psychol. 2019 Aug 16;10:1854. doi: 10.3389/fpsyg.2019.01854 (PMC6707138; doi:10.3389/fpsyg.2019.01854)
Supplement: Supplementary file 1 [file Data_Sheet_1.docx]

**Appendix A: Sitting on Public Transportation**

Some women shared typical behavior. Mary (NS-NW) took a seat alone and remained in the same place when someone came along. Margaret (NS-NW), Carol (NS-OW), and Carla (NS-OW) took a folding seat next to someone without hesitation. Sarah (S-OW-8) took a seat in a row of two and Suzan (S-OW-8) took a folding seat, both near someone.

All other women with obesity, regardless of whether they had or no bariatric surgery, showed atypical behaviors. Dorothy (NS-O1) and Laura (NS-O3) entered a metro car that was completely empty. Dorothy choose a seat that was on its own at the very end of the car. She explained in the RIW that she did not want to see anyone or be seen in the metro and wanted to be alone. Dorothy looked at her phone and “played” when people arrived to “look consistent.” Laura also chose an isolated place and commented: “Well, uh, yes a special place, where I have space with plenty of room, where I won’t crush [anyone]”̇She says people will not dare sit next to her “because if I take up more space than... Because I overflow the seat ... There are people who come to sit down and then they realize that ultimately it doesn’t suit them, and they go and sit somewhere else.” Anita (S-O1-3) and Ana (S-O1-3) sat on a folding seat alone, even when it was not comfortable because they “overflow” the seat. Ana commented: “it’s a habit that I’ve picked up...” Linda (NS-O3) used a strategy of being alone when the car was empty. She sat on three empty seats, but in the RIW she commented that she rushed to a specific seat, the farthest seat in a row of four seats. This was the result of calculations and placement strategies at the door, to be the first in the car to be able to run to the right place where she could “encroach on an empty space without encroaching on the other side.” Her position was uncomfortable because she sat on only one buttock, but she said, “I prefer to hurt myself rather than disturb others.” For Deborah (NS-O1), see the mean text.

The same pattern of behaviors was shown both by women with overweight status who had bariatric surgery (4 months ago) and those who had no surgery. Kerry (S-OW-4) looked for isolated seats and said, “Instinctively, I’ll always look for uh ... somewhere where I’m all alone, not to take the risk of bothering others. When I sat beside someone... hmm... the person was ... curling up, (...) ... I think about it all the time, when I sit down. (...) and so, I anticipate, what will happen. In that seat, I’m, well, I’m secure, I... I’m fine, there’s nobody next to me, so I... I see nobody, I look at nobody, nobody looks at me, no ... It’s, uh ... No, I like... I like this seat.” Karen (S-OW-4) did not take the metro because “it stresses her,” in fact, all public transportation does. She explained her strategy that allows her not to be in too close contact with other people. Also, in light of her explanations, we consider her behaviors atypical.

**Appendix B: Going through Turnstiles and Doors**

Women with obesity and one women with overweight status who had surgery (Dorothy (NS-O1), Deborah (NS-O1), Laura (NS-O3), Linda (NS-O3), Ana (S-O1-3), Anita (S-O1-3), and Kerry (S-OW-4) went through turnstiles and doors sideways, even though they could have easily gone through frontways. In the RIW, Kerry commented: “I think it’s a reflex ... well, I’ve always done that, uh ... Because I used to take up space ... and I think it stayed with me, that’s all.” With Karen (S-OW-4), no situation could be observed as she did not want to take the metro and said that it brought back bad memories. Ana (S-O1-3) also commented: “Well, that’s true, [my] tendency to go through doors sideways, because I don’t realize what the dimensions are. It’s true we won’t allow ourselves to be ridiculed, we know we’re beefy and so, we won’t ... Now, I don’t have an accurate eye, I can’t really know, obviously, I won’t go through frontways if I can’t get through. If I try frontways and can’t get through, I have to go back to go through sideways, and get in the way, and if the [next] person thinks I can go through, and if I don’t go through, in case she bumps into me, that would be embarrassing, I would feel embarrassed and ridiculed.” We did not observe such behavior with the two women with normal weight (Mary, NW, and Margaret, NW), women with overweight status (Carol, NS-OW, and Carla, NS-OW), and the long-term ex-obese women (Suzan, S-OW-8 and Sara, S-OW-8), yet Sara mentioned that before her surgery she used to go through doors and turnstiles sideways.

**Appendix C: Navigating Public Spaces**

Persons with normal weight and overweight status sometimes pass other people who walk more slowly. Mary (NS-NW), Margaret (NS-NW) Carol (NS-OW), and Carla (NS-OW) walked fast, took turns very close to walls in metro corridors, and entered narrow passages such as a small space between a man and a wall quickly and without hesitation.

In our sample, women with obesity and overweight status who did not had bariatric surgery tended to let others pass by when crossing paths (by slowing down, deviating from their course, or even stopping). They tended to walk more slowly and not engage in agile maneuvers. In the RIWs, they mentioned their slower and more cautious pace when navigating. Deborah (NS-O1), Dorothy (NS-O1), Linda (NS-O3), and Laura (NS-O3) tended to deviate from their course when crossing paths with someone, or give way. In the RIW, Laura said, “I make sure that when they pass by, they’re not embarrassed by me.” Linda let everybody in the metro get out of the car before her. That is very striking. She commented in the RIW with many gestures about the metro’s conveyor belts in long corridors: “I stay next to the pole when I hear the footsteps of people behind, either I step aside so they can pass, or I let the entire crowd pass, and once the crowd has passed I go.” Concerning women with obesity who had bariatric surgery, results depended on the participant. Anita (S-O1-3) and Ana (S-O1-3) tended to deviate when crossing paths, as was visible because their trajectory was not completely straight. Anita said: “I step aside; it’s a reflex reflex to avoid interference with those who come towards me. Even if we have changed, we still keep certain habits that ... That are ... Well, precisely, as we take up a bit more space, well, we go into a corner, so we don’t bother [others].” The two women who had never been individuals with obesity also showed atypical behavior. Ana said: “I move aside, it’s an automatic reflex in order not to obstruct those coming towards me.” Another example: Karen (S-OW-4) was walking. A woman was in her way. She could have made a detour through another lane, but she said to the woman, “Excuse me, madam,” and passed by as the woman gave way; then Karen passed through the rather narrow space between a girl with the ice cream and a shelf, displaying typical behavior. Suzan (S-OW-8) showed the same pattern of typical behavior: she passed by a few people, got close to walls, was not passed by anyone, and made no particular attempt to give way. Sarah’s (S-OW-8) behavior was in-between: “It depends. In general, I try to give way, but I do sometimes force my way through because sometimes I have to, but most of the time I give way. Sometimes I do it, when I’m really in a hurry, but otherwise ... It’s not worth it.” She explained that she preferred to avoid conflict and adopted the same approach that she had before her operation. For Kerry (S-OW-4), see the mean text.

**Appendix D: Choosing Clothing Size**

Mary (NS-NW) and Margaret (NS-NW) picked the right size immediately and did not even bother to take several sizes into the fitting room. For the other participants, choosing the right size seemed difficult. Deborah (NS-O1) tried on two coats. The first coat looked too big. The second was a 50 and fitted. However, her size is usually 48, so she picked sizes that were too big to start with. Dorothy (NS-O1) tried on a coat that was too big. She commented: “That’s to say, for once, there were only large sizes and I should have had taken, uh ... smaller ones, so there, suddenly, I think I took a 48 ... something stupid like that. But I should have picked a 46.” Linda (NS-O3) and Laura (NS-O3) took the largest size they could find. Linda commented: “I know I dress much too large. I could dress less large.” Ana (S-O1-3) and Anita (S-O1-3) explained the same difficulties. Anita explained that sometimes, “some clothes I can take in many different sizes, 54, 56, 52, it depends on how I think, ah, how the clothes are cut.” Similar patterns of behavior were found in participants with overweight. Carla (NS-OW) and Carol (NS-OW) picked two sizes of coat to try on. Carol stated, “Sometimes I take two sizes in the same item. When I put on the first, I think it’s the larger size and I feel bad, so I try on the second and ... I already see that there is a difference, and in fact it was because the first I had tried on was too tight. So, it was not the bigger size, in fact.” Suzan (S-OW-8) said she used to try on clothes that were too small when she was a woman with obesity. She still expressed difficulty in judging her size: “I always go for - I don’t know why - the shelves which don’t have my size [any more] but I go there all the time. Before it was 48, now I think it’s 40-42, I don’t know any more, I have to try them on every time.” Sara (S-OW-8) said, “So I took both sizes as I never know if it’s going to be a size bigger or smaller. There I tried on 44, it was too small, it was really too small, so the 46 was better.” Sometimes participants do not realize they have the wrong size. Kerry (S-OW-4) said, “I tried two different sizes and in fact both fit. When I went to shop, I had a sweater that was really large and I hadn’t realized; it was when I saw [myself in the mirror in] the [subcam] recording that I did [realize] ... It was hanging down in the front, it was, it was horrible ... Horrible. That one I got rid of!” For Karen (OW-4), see the main text.
